# Establishment of an Immortalized Porcine Alveolar Macrophage Cell Line That Supports Efficient Replication of Porcine Reproductive and Respiratory Syndrome Viruses

**DOI:** 10.3390/pathogens13121026

**Published:** 2024-11-21

**Authors:** Nguyen Van Diep, Yuiko Hayakawa-Sugaya, Shingo Ishikawa, Hiroaki Kawaguchi, Yasuo Suda, Mana Esaki, Kosuke Okuya, Makoto Ozawa

**Affiliations:** 1 Joint Faculty of Veterinary Medicine, Kagoshima University, Kagoshima 890-0065, Japan; diep.ngv@gmail.com (N.V.D.); kokuya@vet.kagoshima-u.ac.jp (K.O.); 2 IDEAS Swine Clinic, Asahi 289-2505, Japan; hayakawa-ideas@fk9.so-net.ne.jp; 3 Institute of Veterinary Science, Osaka Metropolitan University, Izumisano 598-8531, Japan; s-ishikawa@omu.ac.jp; 4 Laboratory of Veterinary Pathology, Kitasato University School of Veterinary Medicine, Towada 034-8628, Japan; hirok@vmas.kitasato-u.ac.jp; 5 Graduate School of Science and Engineering, Kagoshima University, Kagoshima 890-0065, Japan; ysuda@sudxbiotec.jp; 6 Joint Graduate School of Veterinary Medicine, Kagoshima University, Kagoshima 890-0065, Japan; k0068103@kadai.jp

**Keywords:** porcine reproductive and respiratory syndrome virus, porcine alveolar macrophage, immortalization

## Abstract

Porcine reproductive and respiratory syndrome (PRRS), which is caused by the porcine reproductive and respiratory syndrome virus (PRRSV), has a significant impact on the global pork industry. It results in reproductive failure in sows and respiratory issues in pigs of all ages. Despite the availability of vaccines, controlling the PRRSV remains challenging, partly owing to the limitations of cell culture systems. Current methods largely rely on primary porcine alveolar macrophages (PAMs), which must be harvested from piglets and have limited proliferative capacity. Although some simian cell lines support PRRSV replication, their inability to express porcine CD163, which is a key receptor for PRRSV entry, compromises their effectiveness, because the virus replicates differently in these non-target cells. To address these issues, we established an immortalized PAM cell line, PAM-T43, using SV40 large T antigen for immortalization and porcine serum as a culture supplement. PAM-T43 cells maintain essential macrophage functions, including CD163 expression and phagocytic activity, and exhibit high sensitivity to the PRRSV, efficiently supporting viral replication. This novel cell line offers significant potential for advancing PRRSV research, particularly in vaccine development and field strain isolation, by overcoming the limitations of current systems.

## 1. Introduction

Porcine reproductive and respiratory syndrome (PRRS) is one of the most serious diseases that affect the pork industry worldwide. This is because it causes reproductive failure in sows and complex respiratory syndrome in pigs of all ages [1]. The economic losses to the pork industry caused by PRRS have been estimated at USD 6.25–15.25 per pig in North America and Europe [2,3], and the rate is even higher in Asia [1]. The etiological agent, the porcine reproductive and respiratory syndrome virus (PRRSV), is an enveloped positive-sense single-stranded RNA virus belonging to the family *Arteriviridae* of the order *Nidovirales* [4]. Although both live attenuated and killed vaccines against the PRRSV are available, it remains out of control in most pig farms.

One of the difficulties faced in PRRSV research is the limitation of available cell culture systems. Because PRRSV antigens are mainly detected in cells of the monocyte/macrophage lineage in vivo, primary cultures of porcine alveolar macrophages (PAMs) are commonly used for the isolation and propagation of PRRSV in vitro [5,6]. Although this primary cell culture system has provided a useful model for PRRSV research, PAMs are generally harvested from sacrificed piglets [7] by means of costly, complicated, and time-consuming methods. Moreover, there is the risk of contamination with other pathogens infecting piglets. In addition, primary cells have limited growth potential, with batch-to-batch heterogeneity.

Among various established and continuous cell lines, African green monkey (*Chlorocebus sabaeus*) fetal kidney-derived MA-104 cells and their derivatives, i.e., Marc-145 and CL2621 cells, support PRRSV replication [8,9]. However, these cell lines neither originate from pigs nor belong to the monocyte/macrophage lineage. More importantly, the cells do not express the monocyte/macrophage surface glycoprotein CD163, which is a critical cellular receptor for the infection of PAMs by PRRSV [10]. Consequently, the PRRSV replication mode in these simian cells differs from that in the authentic target cells. In fact, one of the live attenuated vaccine strains, JJ1882, was established by serial passages of VR2332, a virulent isolate strain [11].

These disadvantages of the primary PAM and simian cell lines have hampered PRRSV research. Because PAM-based continuous cell lines potentially offer a solution to these issues, several attempts have been made to immortalize PAMs. Weingartl et al. first established three cell lines of immortalized PAMs by transfecting primary PAMs with a plasmid expressing the oncogenic simian virus 40 large T antigen (SV40T) [12]. However, none of these cell lines supported PRRSV replication or expressed detectable levels of CD163. Although several porcine monocytic/macrophage cell lines have subsequently been described [13,14,15,16,17,18,19,20], the susceptibility of these cell lines to strains of PRRSV, especially field strains, has not been well characterized.

In the present study, we transduced primary PAMs with a retrovirus vector for the expression of SV40T in a porcine serum-containing medium and successfully established an immortalized PAM cell line. Cytological and virological characterizations revealed that the established cell line PAM-T43 is useful for PRRSV research.

## 2. Materials and Methods

### 2.1. Cells

Primary PAMs were obtained from two-to-three-week-old piglets, as described by Wensvoort et al. [7], according to protocols approved by the Animal Care and Use Committee of Kagoshima University (approval number: MD18031). The harvested cells were suspended in cryopreservation medium CELLBANKER 1plus (Zenoaq Resource Co., Ltd., Koriyama, Japan) and stored at −80 °C until required. Primary PAMs and PAM-derived cells were grown in RPMI1640 medium supplemented with 2 mM l-glutamine, 10% fetal bovine serum (FBS), and antibiotics, unless indicated otherwise. African green monkey fetal kidney-derived MA-104 cells, which were provided by Dr. Akira Taneno (Vaxxinova Japan, Tokyo, Japan), and porcine kidney-derived PK-15 cells were grown in Eagle’s Minimum Essential Medium supplemented with 2 mM l-glutamine, 10% FBS, and antibiotics (100 U/mL penicillin, 100 μg/mL streptomycin, and 25 μg/mL amphotericin B). Platinum-GP cells (Cell Biolabs, Inc., San Diego, CA, USA), which are packaging cells for pantropic murine leukemia virus-based retrovirus vectors, were grown in Dulbecco’s modified Eagle’s medium supplemented with 10% FBS, 10 µg/mL blasticidin, and antibiotics. All cells were cultured at 37 °C in 5% CO_2_.

### 2.2. PRRS Viruses

Two live attenuated type-2 PRRSV vaccine strains, i.e., Ingelvac PRRS MLV (Ingelvac; Boehringer Ingelheim Animal Health Inc., Ingelheim, Germany) and Fostera PRRSV (Fostera; Zoetis Inc., Parsippany, NJ, USA), were purchased as commercial products and propagated in MA-104 cells to make virus stocks. Two Japanese type-2 PRRSV field strains, i.e., KU-PYH-9/15 (PYH-9; Genbank accession number MT796277) and KU-27-156K (156K; Genbank accession number MT796281), were isolated from pig specimens using primary PAMs [21] and propagated in MA-104 cells. According to the open reading frame (ORF) 5-gene nucleotide sequence-based previous classification [22], the Ingelvac, Fostera, PYH-9, and 156K strains belong to clusters II, I, III, and unclassified, respectively [21]. A live attenuated type-1 PRRSV vaccine strain, i.e., UNISTRAIN (HIPRA; Girona, Spain), was purchased as a commercial product and propagated in primary PAMs to make a virus stock. All the PRRSV strains were stored at −80 °C until required.

### 2.3. Generation and Transduction of Retrovirus Vector

The complementary DNA for SV40T was cloned into the multicloning site of the murine leukemia virus-based retrovirus vector pMXs (Cell Biolabs, Inc.). The resultant plasmid, pMXs-SV40T, and the expression plasmid for the G glycoprotein of vesicular stomatitis virus, pCMV-VSV-G (Cell Biolabs, Inc.), were co-transfected into Platinum-GP cells in a 6-well plate with TransIT-293 reagent (Mirus, Madison, WI, USA), according to the manufacturer’s instructions. At 48 h post-transfection, culture supernatants containing the retrovirus vector for the expression of SV40T, i.e., RV/SV40T, were harvested, clarified using a 0.23 µm filter, and stored at −80 °C. Primary PAMs in a 6-well plate were transduced with RV/SV40T by replacing the culture supernatant with the fresh growth medium containing RV/SV40T (5-fold dilution).

### 2.4. Proliferation Kinetics of Cells

Primary PAMs, MA-104 cells, and PAM-T43 cells were seeded in 6-well plates at a density of 1 × 10^5^ cells/well. The cells were trypsinized daily for 6 days, stained with Trypan Blue, and counted and sized using a LUNA-II automated cell counter (Logos Biosystems, Dongan-gu, Anyang-si, Republic of Korea).

### 2.5. Immunofluorescence Assay (IFA)

The expressions of porcine CD163, SV40T, and PRRSV N protein in the cultured cells were detected by IFA. Briefly, the cells were washed with phosphate-buffered saline (PBS) and fixed with Mildform 10N (FUJIFILM Wako Pure Chemical, Osaka, Japan) for 30 min. The fixed cells were washed with PBS and permeabilized with PBS containing 1% bovine serum albumin (BSA) and 0.5% Triton X-100 for 15 min. The permeabilized cells were washed with PBS and incubated with primary antibodies (see below for details) diluted with PBS containing 1% BSA for 1 h at room temperature. After washing with PBS three times, the cells were further incubated with DyLight488- or DyLight594-labeled goat anti-mouse secondary antibody (Abcam, Cambridge, MA, USA) diluted at a ratio of 1:1000 with PBS containing 1% BSA. The cells stained with the secondary antibody were washed with PBS three times and examined using an Eclipse Ti-S fluorescent microscope (Nikon, Tokyo, Japan). The primary antibodies used to detect porcine CD163, SV40T, and PRRSV N protein were mouse monoclonal antibodies 2A10/11 (1:1000 dilution; Bio-Rad, Hercules, CA, USA), PAb416 (1:1000 dilution; Merck KGaA, Darmstadt, Germany), and M10-7 (1:3000 dilution; prepared in our laboratory), respectively.

### 2.6. Virus Titration

Virus titers were determined by means of the 50% tissue culture infectious dose (TCID_50_) assays in the primary PAMs, MA-104 cells, or PAM-T43 cells. Briefly, cell monolayers (2 × 10^4^ cells/well) in 96-well plates were inoculated with 10-fold serial dilutions of PRRSVs in growth medium (four wells for each dilution) and cultured for 7–9 days. The infected wells containing the MA-104 and PAM-T43 cells were evaluated by determining the cytopathic effect after crystal violet staining. Because the cytopathic effect in the primary PAMs was occasionally not obvious, the infected wells were evaluated by observing IFA-based PRRSV N protein expression (Supplementary Figure S1). Virus titers were calculated using the Reed–Muench method [23].

### 2.7. Replication Kinetics of PRRSVs

Cell monolayers (2 × 10^5^ cells/well) in 12-well plates were inoculated with individual PRRSV strains at a multiplicity of infection of 0.01. This was followed by virus adsorption at 37 °C for 2 h. The inoculated cells were washed twice with PBS and cultured with growth medium for 6 days. The cell culture supernatants (50 μL/day) were collected each day for 6 days and stored at −80 °C. The virus titers in the supernatants were determined by TCID_50_ assays in the MA-104 cells, as described above.

### 2.8. Phagocytosis Assay

The PAM-T43 and PK-15 cells (5 × 10^5^ cells) were treated with a pH-sensitive fluorescent dye, i.e., pHrodo Green *E. coli* BioParticles Conjugate (Thermo Fisher Scientific, La Jolla, CA, USA), and seeded in a 24-well plate. The cells were then incubated at either 4 or 37 °C for 1 h. Phagocytosis-associated green fluorescent signals were observed under the fluorescent microscope and analyzed using a BD Accuri C6 Plus flow cytometer (BD Biosciences, Franklin Lakes, NJ, USA).

### 2.9. Flow Cytometric Analysis

Surface expression of porcine CD163, CD14, CD45, CD206, and F4/80 on PAM-T43 cells were analyzed using a flow cytometer. Briefly, trypsinized PAM-T43 cells were washed with PBS and resuspended in PBS containing 1% BSA. The cells were incubated with primary antibodies (see below for details) diluted in PBS containing 1% BSA for 1 h at 4 °C. This was followed by incubation with DyLight594-labeled goat anti-mouse or DyLight488-labeled goat anti-rabbit secondary antibody (Abcam), diluted at a ratio of 1:500 with PBS containing 1% BSA for 1 h at 4 °C. After staining with the secondary antibody, the cells were washed twice with PBS and analyzed on an RF-500 flow cytometer (Sysmex Corporation, Kobe, Japan). The primary antibodies used were as follows: anti-porcine CD163 (mouse monoclonal antibody 2A10/11, 1:100 dilution) and rabbit polyclonal antibodies for CD14 (17000-1-AP, 1:200 dilution; Proteintech Group, Inc., Rosemont, IL, USA), CD45 (20103-1-AP, 1:50 dilution; Proteintech Group, Inc.), CD206 (18704-1-AP, 1:200 dilution; Proteintech Group, Inc.), and F4/80 (28463-1-AP, 1:100 dilution; Proteintech Group, Inc.).

## 3. Results

### 3.1. Establishment of CD163-Expressing Immortalized PAM

To establish PAM-derived cell lines that are highly susceptible to PRRSV field strains, we initially transduced primary PAMs with a retrovirus vector for the expression of SV40T (RV/SV40T) in the standard manner and obtained continuously dividing cells. However, these cells did not support the replication of representative PRRSV field strains, likely owing to the limited or absent expression of CD163 in individual cells (Supplementary Figure S2). In a previous study [12], the expression of several immune cell surface markers in the immortalized PAM was upregulated by supplementation with porcine serum, although the effect of porcine serum on CD163 expression was not evaluated. Therefore, we next transduced primary PAMs with RV/SV40T in growth medium, additionally supplemented with 10% porcine serum, and obtained continuously dividing cells. In contrast to the immortalized PAMs established without porcine serum, the PAMs immortalized in the porcine serum-containing medium expressed CD163 (Supplementary Figure S2). These results suggest that the porcine serum contributed to the stable expression of CD163 in the immortalized PAMs. Following cell cloning, we established the cell line PAM-T43. PAM-T43 cells that had been passaged 5–10 times after cloning were used in all subsequent experiments, unless otherwise specified.

### 3.2. Cellular Characteristics of PAM-T43 Cells

**Figure 1:**
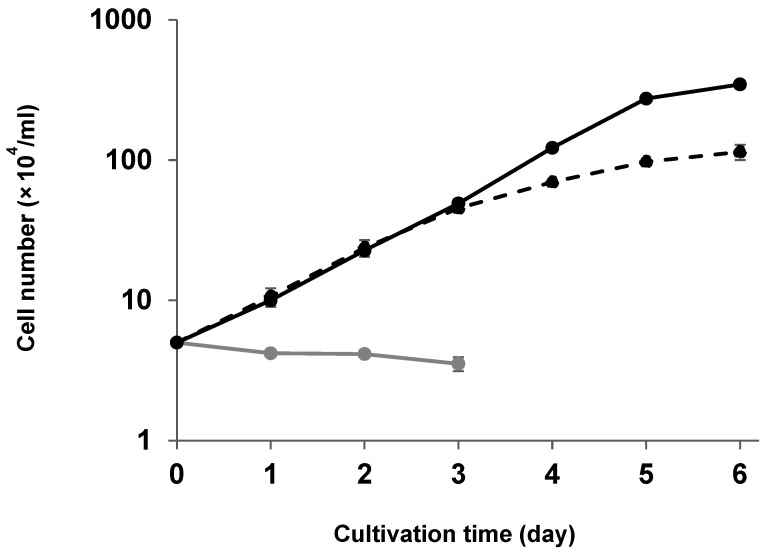
Proliferation kinetics of cells. Primary PAMs (gray line), MA-104 cells (dashed black line), and PAM-T43 cells (black line) seeded in 6-well plates at a density of 1 × 10^5^ cells/well were counted each day for 6 days. Data are presented as mean ± the standard deviation of three independent replicates. (PAMs = porcine alveolar macrophages; PRRSV = porcine reproductive and respiratory syndrome virus.).

To evaluate the proliferation characteristics of PAM-T43 cells, we analyzed the proliferation kinetics. The proliferation kinetics of the PAM-T43 cells were comparable to those of African green monkey-derived MA-104 cells, which are the gold standard cell line for PRRSV propagation (Figure 1). By contrast, the number of primary PAMs gradually decreased over the testing period. These results indicate the efficient proliferation characteristics of PAM-T43 cells.

To confirm the expression of SV40T in the PAM-T43 cells, the cells were subjected to immunofluorescence assays with an SV40T-specific antibody (Supplementary Figure S3). MA-104 cells transiently transfected with pMXs-SV40T and primary PAMs served as positive and negative controls, respectively. Fluorescent signals originating from the SV40T were detected in most of the PAM-T43 cells and a limited portion of the plasmid-transfected MA-104 cells, but not in the primary PAMs. These results suggest that the immortalization of PAM-T43 cells can be accounted for by the stable expression of SV40T.

To determine whether PAM-T43 cells inherit the characteristics of macrophages, the phagocytic activity was investigated using a pH-sensitive fluorescent dye conjugate as a phagocytic probe. After treatment with the phagocytic probes followed by incubation at 37 °C, clear fluorescent signals were detected in the PAM-T43 cells, but not in the porcine kidney-derived PK-15 cells (Supplementary Figure S4A), suggesting that the phagocytic probes were ingested only in PAM-T43 cells. We also tested the effect of the temperature shift on PAM-T43 cell-specific ingestion by flow cytometric analysis. The proportion of the fluorescent signal-positive cells kept at 4 °C (4.53%) increased to 71.76% after incubation at 37 °C for 1 h (Supplementary Figure S4B). This suggested that ingestion was mediated by temperature-dependent cellular action.

To further confirm the macrophage-like characteristics of PAM-T43 cells, the surface expression of representative monocyte/macrophage-specific markers was examined by flow cytometric analysis. In addition to CD163 (Supplementary Figure S5A), other markers, such as CD14, CD45, CD206, and F4/80 (Supplementary Figure S5B) were also detected on PAM-T43 cells. These findings indicate that PAM-T43 cells inherit the characteristics of macrophages.

### 3.3. Sensitivity of PAM-T43 Cells to PRRSVs

**Figure 2:**
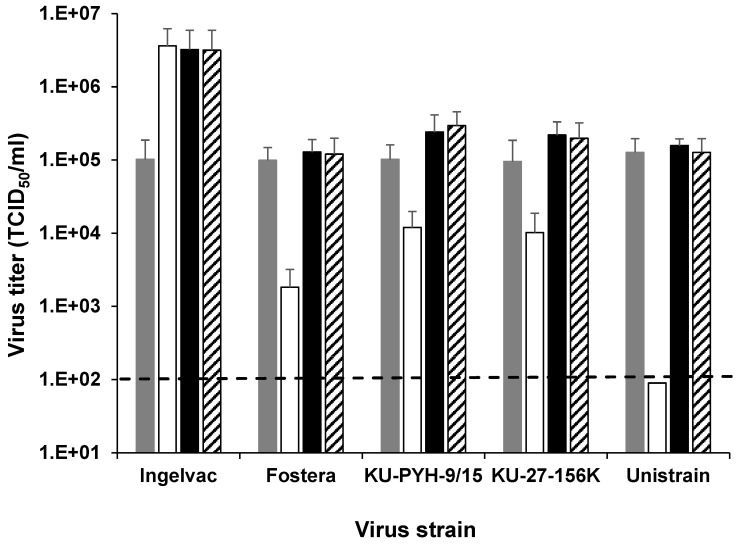
PRRSV sensitivity of cells. Titers of five representative PRRSV strains determined in primary PAMs were adjusted to 10^5^ TCID_50_/mL and redetermined by the TCID_50_ assays in primary PAMs (gray bars), MA-104 cells (white bars), PAM-T43 cells (black bars), and PAM-T43 cells after 100 passages (striped bars). Data are presented as mean ± the standard deviation of three independent replicates. The dashed line indicates the detection limit (1 × 10^2^ TCID_50_/mL). (PRRSV = porcine reproductive and respiratory syndrome virus; PAMs = porcine alveolar macrophages; TCID_50_ = 50% tissue culture infectious dose.).

To evaluate the sensitivity of PAM-T43 cells with regard to PRRSVs, 10^5^ TCID_50_/mL each (determined in primary PAMs) of five representative PRRSV strains were re-titrated by means of a TCID_50_ assay in PAM-T43 cells, primary PAMs, and MA-104 cells (Figure 2). The titers of the Fostera, PYH-9, 156K, and UNISTRAIN strains determined in the PAM-T43 cells were the highest among those in the three cell lines tested. More importantly, the titers of these four PRRSV strains in the PAM-T43 cells were significantly higher than those in the MA-104 cells. Although the Ingelvac strain was established by serial passages in MA-104 cells, the virus titers determined in MA-104 and PAM-T43 cells were comparable. These results indicate that the sensitivity of PAM-T43 cells with regard to the PRRSV field strains is comparable to that of primary PAMs and significantly higher than that of MA-104 cells. Notably, the virus sensitivity of PAM-T43 cells did not change significantly, even after 100 passages (Figure 2), suggesting that the fundamental characteristics of the PAM-T43 cells were stable.

### 3.4. Replication Kinetics of PRRSVs in PAM-T43 Cells

**Figure 3:**
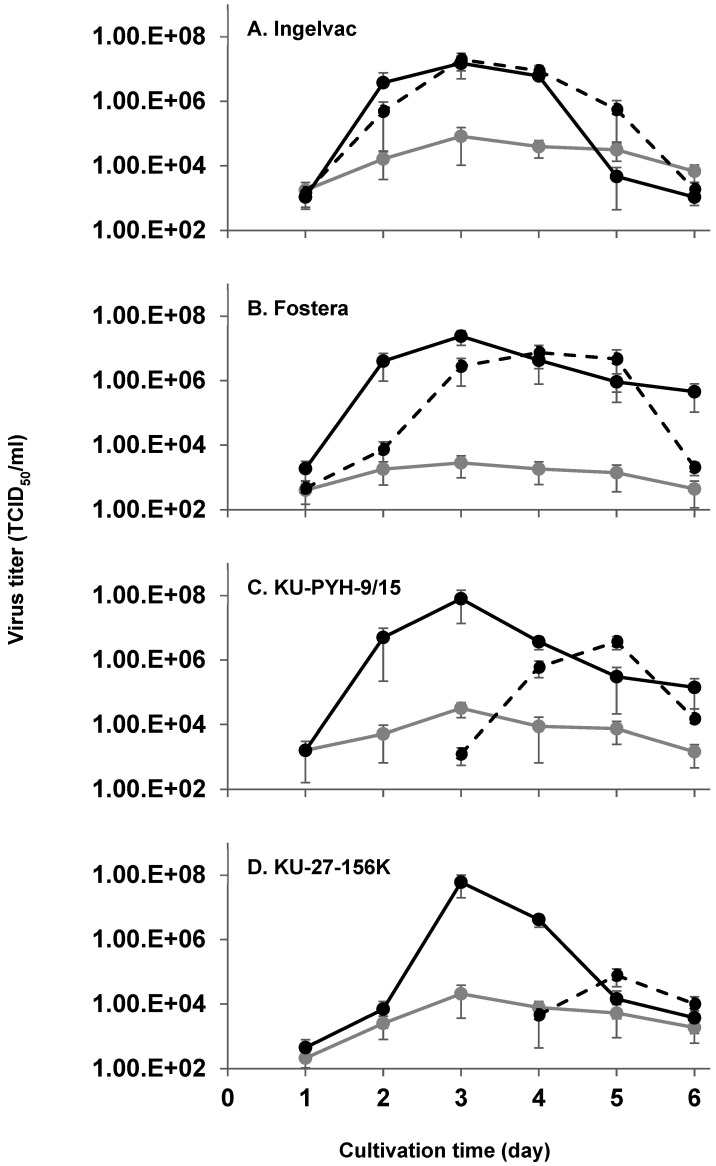
Replication kinetics of PRRSVs in cells. Primary PAMs (gray lines), MA-104 cells (dashed black lines), and PAM-T43 cells (black lines) were inoculated with PRRSV Ingelvac (**A**), Fostera (**B**), KU-PYH-9/15 (**C**), or KU-27-156K (**D**) strains at a multiplicity of infection of 0.01. Virus titers in the supernatants collected each day from the inoculated cells were determined by TCID_50_ assays in the MA-104 cells. KU-PYH-9/15 and KU-27-156K strains were not detectable in supernatants from MA-104 cells on earlier days post-inoculation, likely due to their limited replication kinetics. Data are presented as mean ± the standard deviation of three independent replicates. (PRRSV = porcine reproductive and respiratory syndrome virus; PAMs = porcine alveolar macrophages; TCID_50_ = 50% tissue culture infectious dose.).

To evaluate PRRSV replication in the PAM-T43 cells, the replication kinetics of the four representative PRRSV strains in the PAM-T43 cells, primary PAMs, and MA-104 cells, was analyzed (Figure 3). The highest titers of the Fostera, PYH-9, and 156K strains were recorded in PAM-T43 cells on 3 days post-infection. While the highest titer of the Ingelvac strain was recorded in MA-104 cells, the replication kinetics of the remaining three PRRSV strains in MA-104 cells were lower and slower than those in PAM-T43 cells. In particular, the two PRRSV field strains, PYH-9 and 156K, replicated much faster and more prolifically in PAM-T43 cells than in MA-104 cells. The replication kinetics of all the four PRRSV strains in primary PAMs was much less than the counterparts in PAM-T43 and MA-104 cells, likely owing to their limited dividing ability. These results indicate that PAM-T43 cells enable PRRSV field strains to replicate efficiently.

## 4. Discussion

Herein, we describe the establishment of an immortalized PAM cell line (PAM-T43) using SV40T and porcine serum as the immortalization factor and culture supplement, respectively. The PAM-T43 cells propagated efficiently (Figure 1) and expressed SV40T stably (Supplementary Figure S3). Additionally, the PAM-T43 cells inherited the characteristics of macrophages, including phagocytic activity (Supplementary Figure S4) and surface expression of representative monocyte/macrophage-specific markers (Supplementary Figure S5). More importantly, the PAM-T43 cells demonstrated high sensitivity to PRRSV infection (Figure 2) and strong support for PRRSV replication (Figure 3). These results indicate that PAM-T43 cells are useful for PRRSV research, including field strain isolation and vaccine development.

The ORF5 gene of PRRSVs has been widely used as the primary target for phylogenetic classification [24]. This is because its product, glycoprotein 5 [25], is a key component of the viral envelope that has diversified under selective pressure from host immune responses. However, the reliability of ORF5 gene-based classification is limited, primarily owing to the gene’s relatively short length (approximately 600 nucleotides) and the frequent intragenic recombination events among PRRSV strains [26]. Consequently, whole-genome sequencing is considered a more accurate method for the characterization of PRRSV field strains [27,28]. Although next-generation sequencing is necessary to obtain high-resolution whole-genome sequences of PRRSVs from clinical samples, such advanced technology is not always readily accessible. Furthermore, contaminants present in clinical samples can interfere with the sequencing process. To address these challenges, PRRSV isolation in cultured cells, followed by a cost-effective next-generation sequencing approach—such as Nanopore sequencing using a Flongle flow cell (Oxford Nanopore Technologies, Oxford, UK)—offers a straightforward solution. The high sensitivity of PAM-T43 cells to PRRSV infection (Figure 2) facilitates efficient virus isolation, thereby enhancing the likelihood of successful genome sequencing and, consequently, accurate characterization.

Various types of PRRS vaccines are globally available; however, PRRSV remains uncontrolled in most pig farms. Therefore, the development of more effective PRRS vaccines is highly desirable. A key factor in the development of new vaccines is the availability of a robust cell line that can proliferate efficiently and yield high virus titers. The proliferation kinetics of PAM-T43 cells were superior to those of the well-established MA-104 cell line (Figure 1). Additionally, all four PRRSV strains tested in the present study propagated efficiently in PAM-T43 cells (Figure 3). These results suggest that PAM-T43 cells are a promising platform for the development and production of PRRS vaccines.

Our results clearly demonstrate that porcine serum supplementation in the culture medium plays a critical role in maintaining stable CD163 expression in the immortalized PAMs (Supplementary Figure S2). Similarly, Weingartl et al. reported that porcine serum was associated with the upregulation of several immune cell surface markers in immortalized PAMs [12]. However, none of their established cell lines supported PRRSV replication, even in porcine serum-containing medium, likely owing to the limited CD163 expression on their surfaces. In contrast, the exogenous expression of porcine CD163 was necessary to confer PRRSV susceptibility to one of their cell lines, 3D4/21 cells [29,30]. Notably, in the present study, porcine serum was supplemented throughout the entire immortalization and maintenance process, whereas Weingartl et al. introduced porcine serum only after immortalization [12]. Although the precise molecular mechanism remains unclear, our findings suggest that the timing of porcine serum supplementation is crucial for stable CD163 expression. Additionally, our results imply that adjusting the animal species origin of both cell lines and supplemented sera could further enhance the effectiveness of cell culture studies.

In Japan, the vast majority of PRRSV field strains are type-2 viruses [22], with only a single outbreak caused by type-1 PRRSVs recorded to date [31]. Consequently, most of the virus strains tested in the present study were limited to type-2 PRRSVs. This is a limitation of this study. Although our findings with UNISTRAIN, i.e., the live attenuated type-1 PRRSV vaccine strain, suggest the potential applicability of PAM-T43 cells for supporting type-1 PRRSV field strains, a more comprehensive validation study is required.

The high sensitivity of the PAM-T43 cells to infection with two PRRSV filed strains (Figure 2) suggests their potential utility for virus isolation from clinical samples. Although ZMAC cells, an established cell line derived from porcine alveolar macrophages [15], have been shown to support the efficient isolation of a wide range of PRRSV field strains [32], certain cellular characteristics, such as a slow growth rate (doubling timee: ~72 h), may limit their widespread use. In contrast, the rapid propagation of the PAM-T43 cells offers ease of handling and may facilitate broader application.

## 5. Conclusions

We successfully established an immortalized PAM cell line, PAM-T43, and demonstrated its utility in PRRSV research. The cellular characteristics of PAM-T43 cells also suggest their potential for studying other porcine viruses, including African swine fever virus. We believe that this newly developed cell line will contribute significantly to advancements in porcine health research and the pork industry.

## Data Availability

The original contributions presented in this study are included in the article/supplementary material. Further inquiries can be directed to the corresponding author.

## Supplementary Materials

The following supporting information can be downloaded at: https://www.mdpi.com/article/10.3390/pathogens13121026/s1, Supplementary Figure S1: Expression of PRRSV N protein in primary PAMs; Supplementary Figure S2: CD163 expression in cells; Supplementary Figure S3: SV40T expression in cells; Supplementary Figure S4: Phagocytic activity; Supplementary Figure S5: Surface expression of monocyte/macrophage-specific markers on PAM-T43 cells.
