# Supplementary material for: Establishment of an Immortalized Porcine Alveolar Macrophage Cell Line That Supports Efficient Replication of Porcine Reproductive and Respiratory Syndrome Viruses
Source: Pathogens. 2024 Nov 21;13(12):1026. doi: 10.3390/pathogens13121026 (PMC11678208; doi:10.3390/pathogens13121026)
Supplement: Supplementary file 1 [file pathogens-13-01026-s001.zip › pathogens-3298044-supplementary.pptx]

## Slide 1
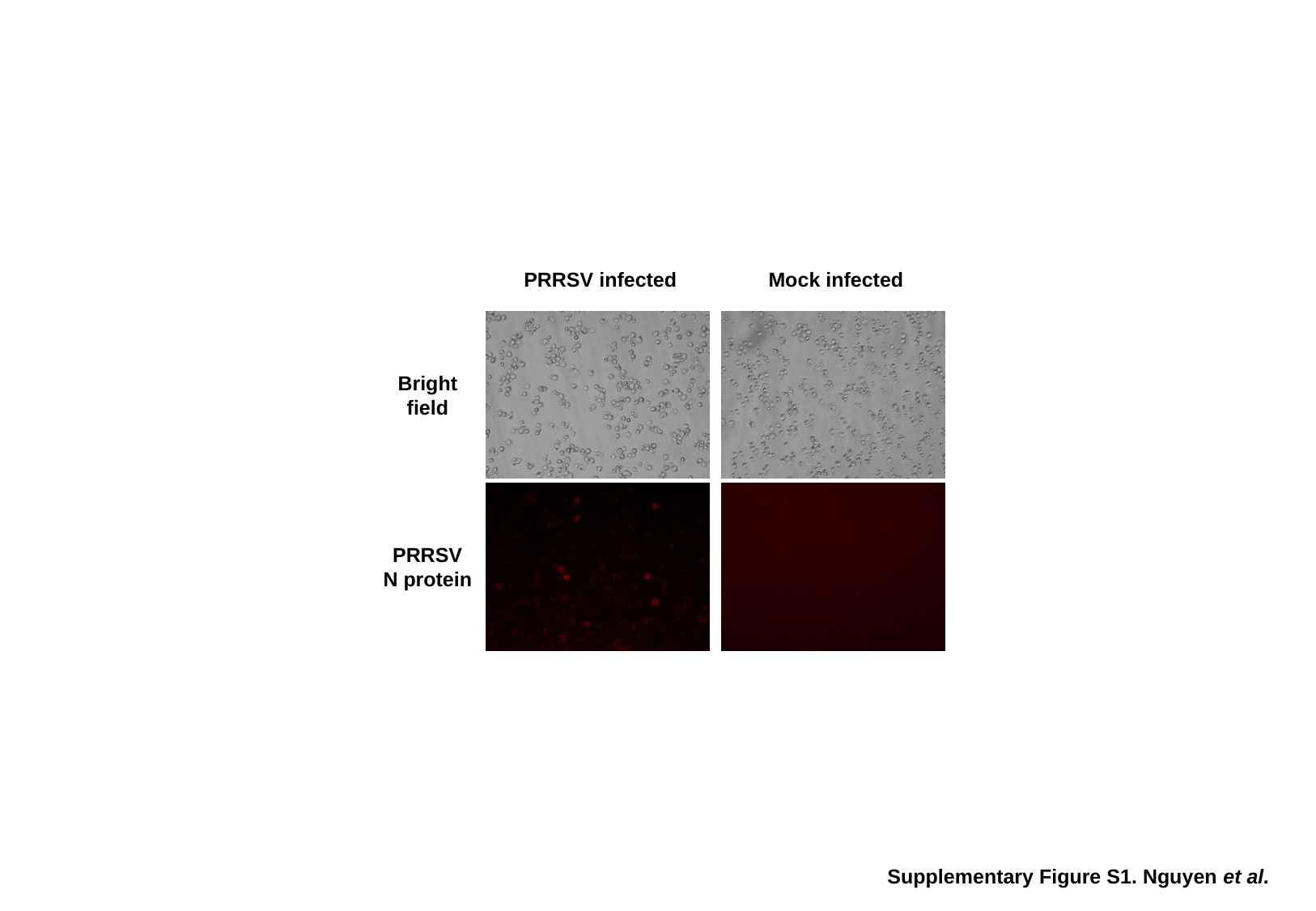

PRRSV infected
 Mock infected
Bright
field
PRRSV
N protein
 Supplementary Figure S1. Nguyen et al.

## Slide 2
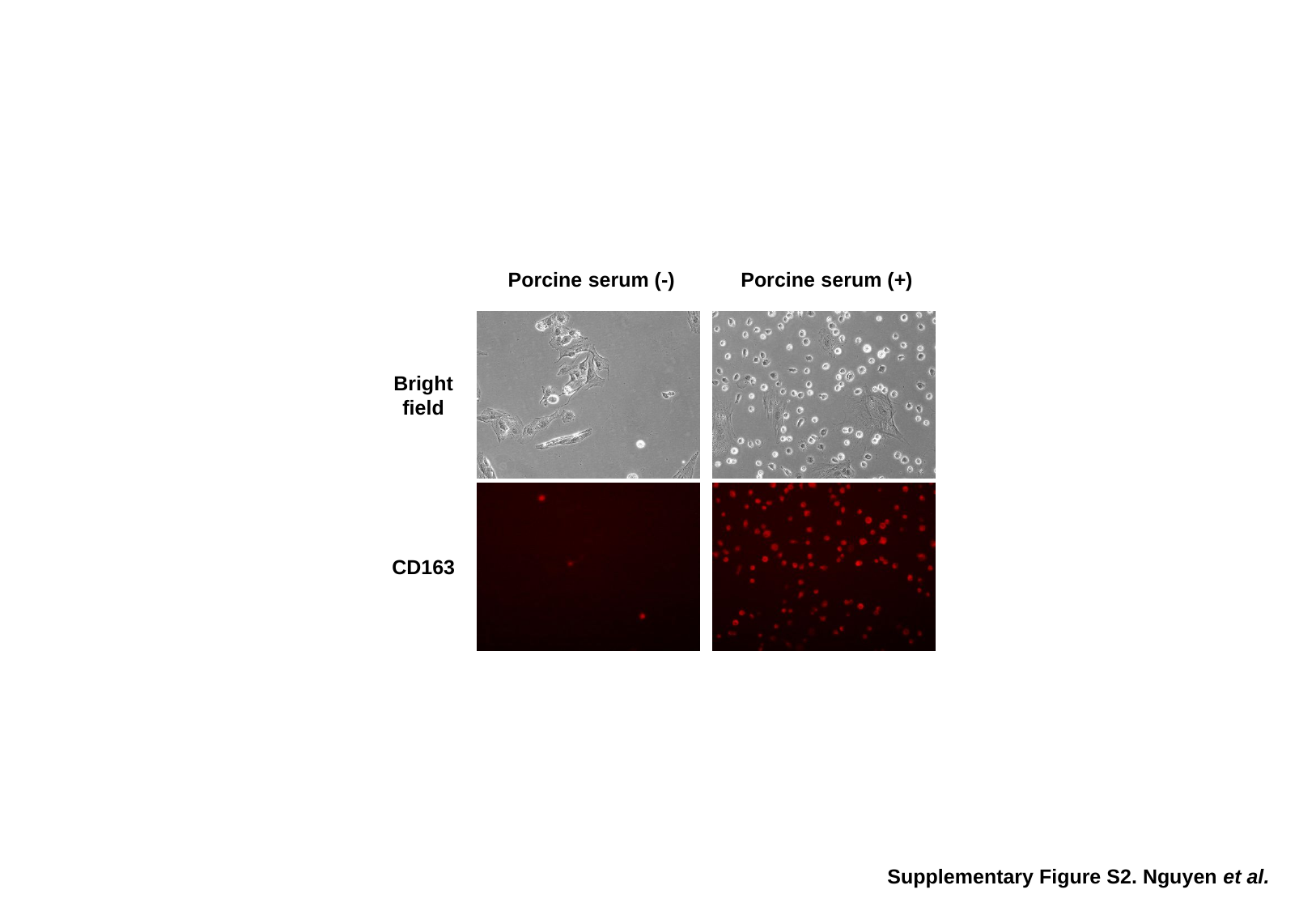

Porcine serum (-)
 Porcine serum (+)
Bright
field
CD163
 Supplementary Figure S2. Nguyen et al.

## Slide 3
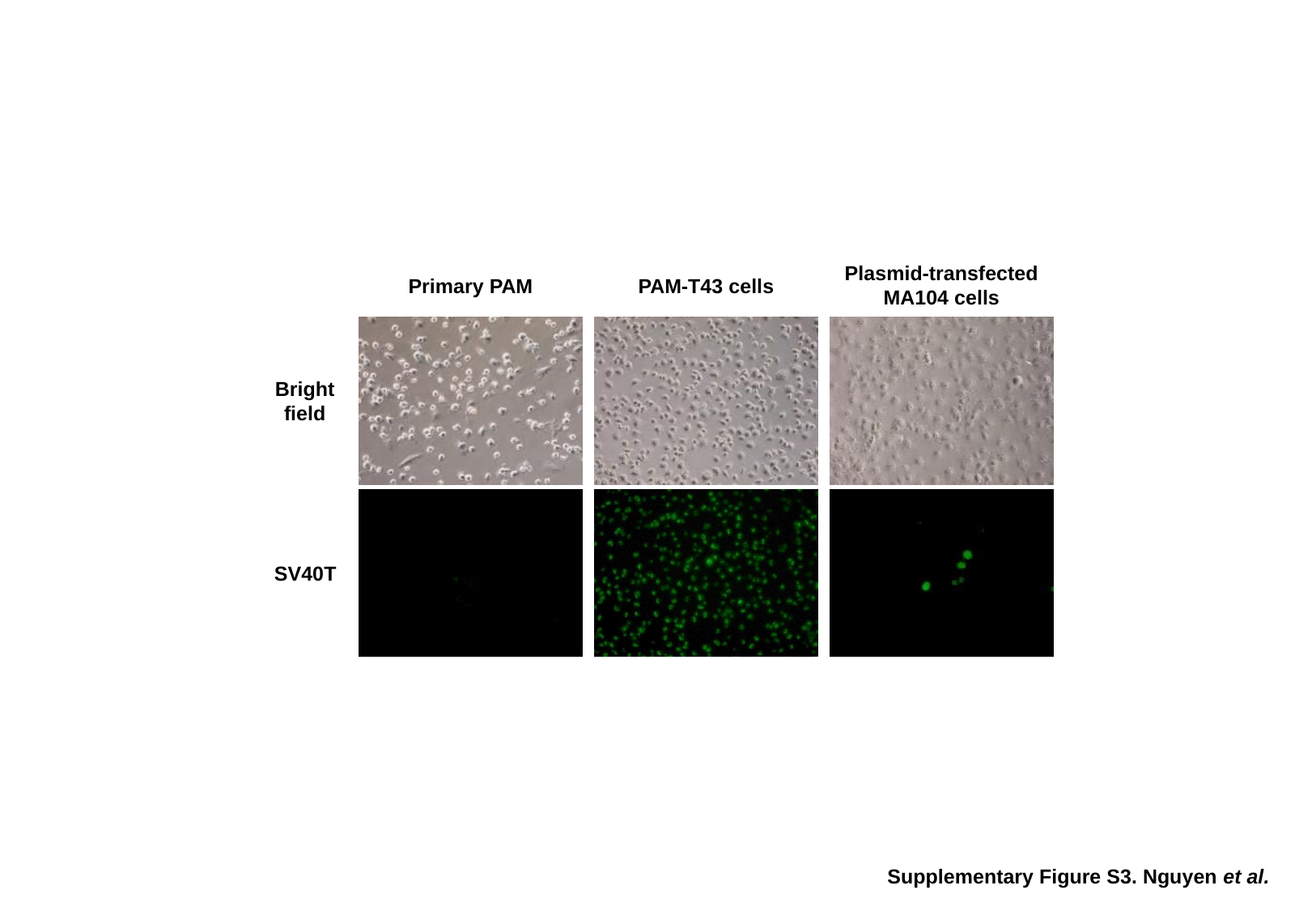

Plasmid-transfected
MA104 cells
Primary PAM
PAM-T43 cells
Bright
field
SV40T
 Supplementary Figure S3. Nguyen et al.

## Slide 4
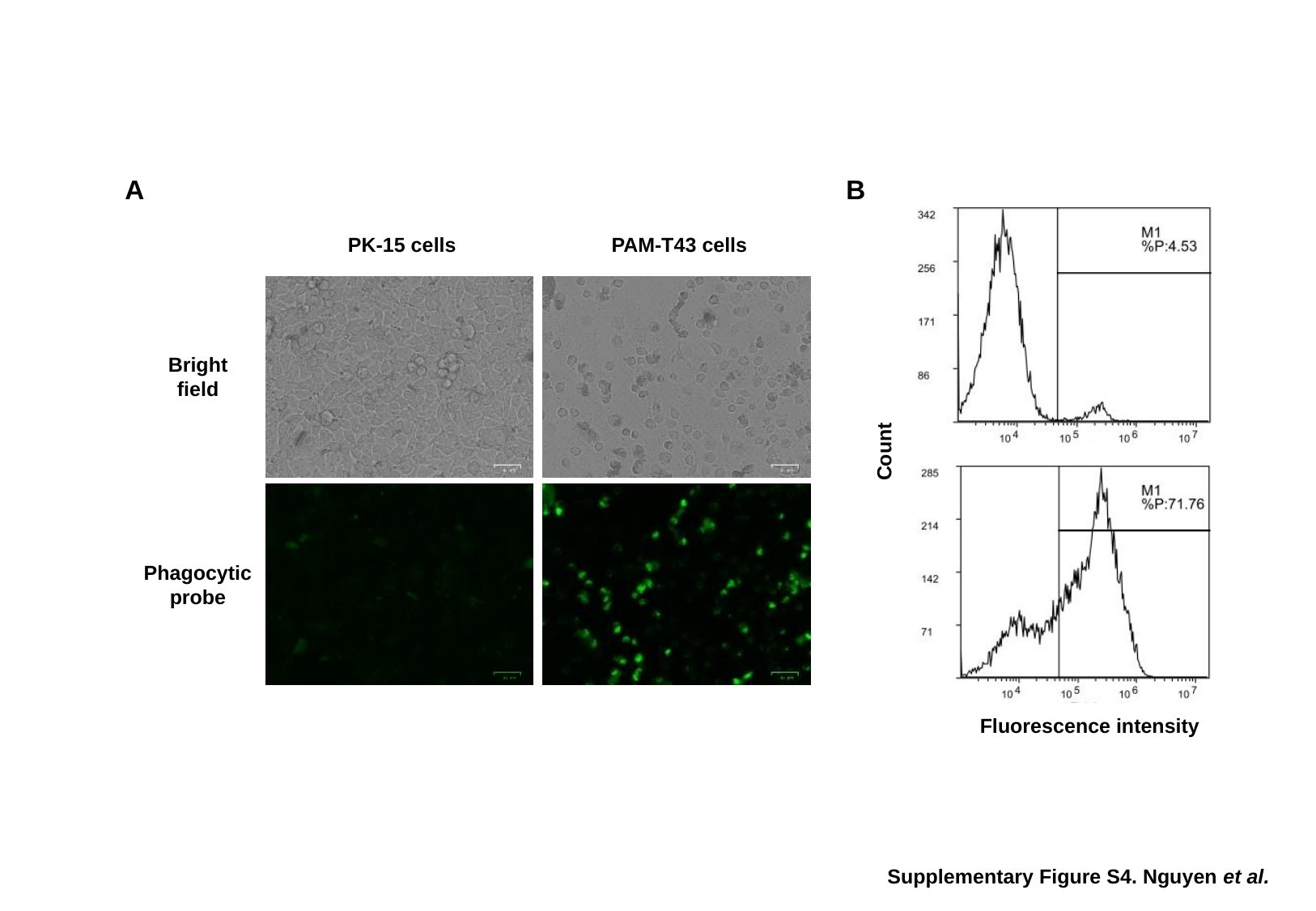

A
B
 PK-15 cells
 PAM-T43 cells
Bright
field
Count
Phagocytic
probe
Fluorescence intensity
 Supplementary Figure S4. Nguyen et al.

## Slide 5
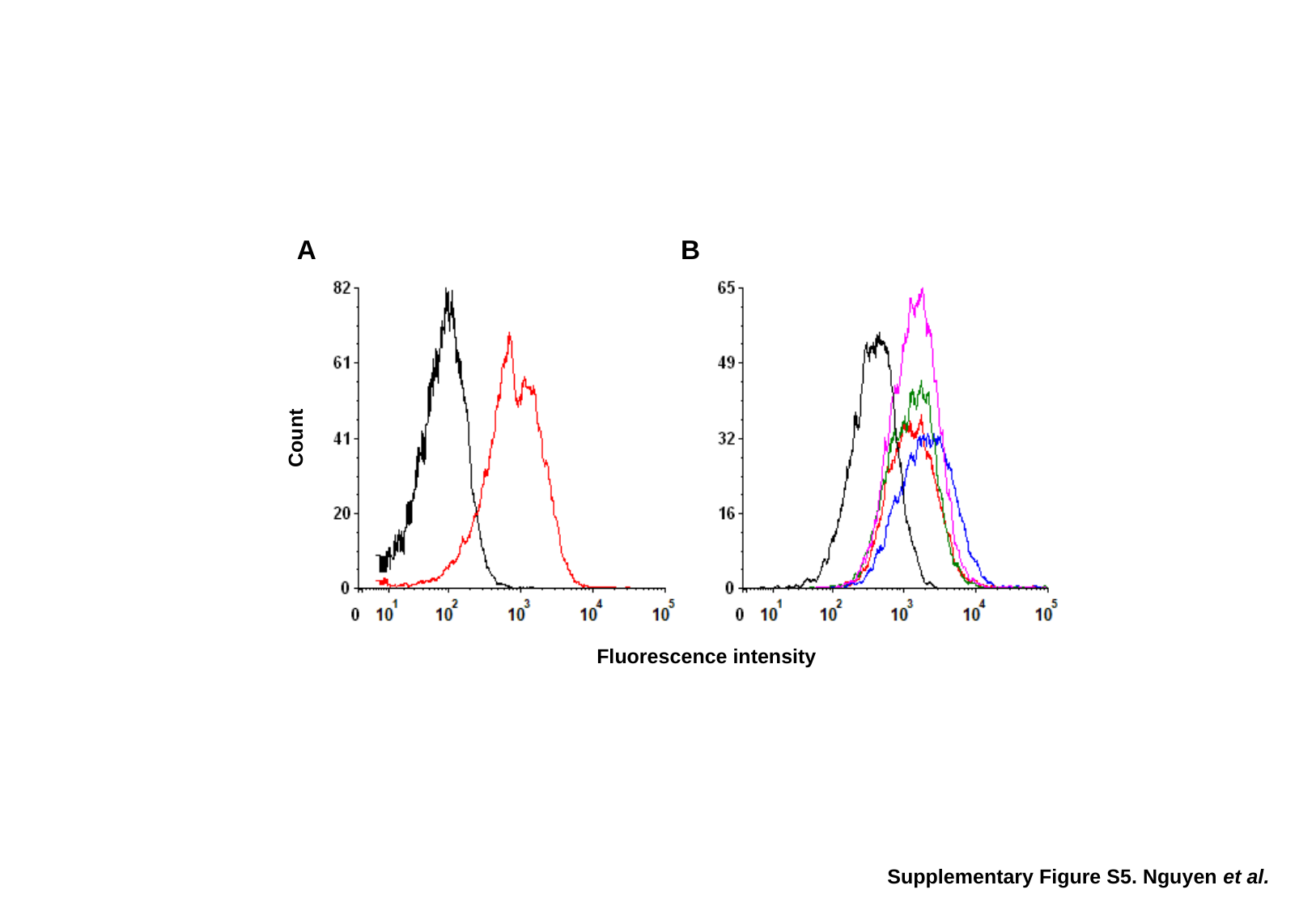

A
B
Count
Fluorescence intensity
 Supplementary Figure S5. Nguyen et al.
